# An Unusual Presentation of a Complicated Urinary Tract Infection With Features Concerning for Malignancy: A Diagnostic Challenge

**DOI:** 10.7759/cureus.107154

**Published:** 2026-04-16

**Authors:** Drew K Knight, Tailla Santos, Amber J Stout, Adrian Martinez Crespo, Ilya Fonarov

**Affiliations:** 1 Internal Medicine, Jackson Memorial Hospital, Miami, USA; 2 Medical School, St. George's University School of Medicine, St. Georges, GRD; 3 Medical School, American University of the Caribbean School of Medicine, Cupecoy, SXM; 4 Medical School, Ross University School of Medicine, Bridgetown, BRB; 5 Internal Medicine, Orlando College of Osteopathic Medicine, Orlando, USA

**Keywords:** bladder stone, complicated urinary tract infection, elevated prostate-specific antigen (psa), fecal incontinence, prostatomegaly

## Abstract

Recurrent urinary tract infections (UTIs) in men are uncommon and frequently signal an underlying pathology. We present the case of a 73-year-old man who presented to the emergency department with a urinary burning sensation, foul-smelling urine, and fecal incontinence when straining to urinate. The patient had a history of recurrent UTIs, benign prostatic hyperplasia (BPH), and prior use of a chronic indwelling urinary catheter. Imaging revealed a 3.5 cm bladder stone, a markedly enlarged prostate with pelvic lymphadenopathy, lumbar vertebral fractures, and a rim-enhancing gluteal collection later confirmed as a hematoma. He improved with intravenous antibiotics and was discharged with plans to follow up as an outpatient for his prostate enlargement, vertebral fractures, and bladder stone management. This case illustrates therapeutic challenges in cases with multiple abnormal findings, as well as the risk of anchoring bias with overlapping clinical entities.

## Introduction

Urinary tract infections (UTIs) are one of the most commonly encountered infections in clinical practice. They can range in presentation, with the classic symptoms being dysuria, urinary frequency, and urinary urgency [1]. Untreated UTIs can ascend through the urinary tract and into the kidneys, where they can invade into systemic circulation and cause life-threatening sepsis. UTIs are a significant cause of sepsis, leading to hospitalization, especially complicated UTIs. Complicated UTIs are defined as any UTI in an immunocompromised patient, pregnant patient, or male patient, as well as any involvement with the kidneys, stones, catheters, urinary obstruction, or fevers [2].

They come with a higher risk of treatment failure, and they can progress quickly, so they should be monitored closely. In patients with complex or overlapping conditions, clinicians must be cautious of anchoring bias, the tendency to prematurely attribute symptoms to a single diagnosis when another pathology may be present [3]. This is particularly important in cases of dual pathology or atypical presentations, where fixation on an initial diagnosis can delay recognition of additional or more serious underlying disease.

In male patients, evaluation of urinary symptoms may sometimes include measurement of prostate-specific antigen (PSA), particularly in those with known benign prostatic hyperplasia (BPH) or other prostate abnormalities. However, PSA interpretation in the setting of an acute UTI requires caution, as levels can be transiently elevated due to acute inflammation or irritation of the prostate. In contrast, chronically elevated PSA may reflect long-standing BPH, prostatic inflammation, or malignancy [4]. Without prior PSA measurements for comparison, the clinical significance of an isolated PSA elevation during an acute infection is limited, and results must be interpreted in the broader context of the patient’s clinical presentation and follow-up evaluation.

## Case presentation

A 73-year-old male presented to the emergency department with a chief concern of fecal incontinence when straining to urinate. He had a past medical history of hypertension, BPH, prior chronic catheterization, and recurrent UTIs. He stated that for two weeks, every time he strained to urinate, he would also pass stool unintentionally. He was experiencing a burning sensation with urination and an occasional foul odor of urine. He complained of not always being able to make it to the restroom before these accidents occurred, and often would need to strain to relieve his bladder. He denied any recent falls, accidents, or trauma. He used to work at a parks and recreation department, but he was presently unemployed and resided at a homeless shelter. He was not on any medications. He denied alcohol, tobacco, and other illicit drug use. 

On the physical exam, he was febrile at 38.6 °C, with a blood pressure of 112/70 mmHg, a pulse of 82 beats per minute, and a respiratory rate of 18 breaths per minute. The musculoskeletal exam was notable for tenderness upon palpation along the lower lumbar area and suprapubic tenderness. Digital rectal examination demonstrated a diffusely enlarged prostate without nodularity and diminished anal sphincter tone. The remainder of the physical exam was unremarkable. Urinalysis revealed 262 white blood cells (WBC). A urine culture was obtained via a mid-stream clean-catch sample and was positive for gram-negative rods. He was put on ceftriaxone 2 g every 24 hours and admitted to the hospital for further evaluation of his symptoms.

The patient was not formally evaluated for urinary retention, as he declined urinary catheterization. Clinically, he improved rapidly after admission and was able to void freely without pain, suggesting no significant urinary retention. Laboratory investigations were notable for elevated white blood cells (WBC) at 13.5 × 10⁹/L, elevated blood urea nitrogen (BUN) at 28 mg/dL, elevated creatinine at 1.9 mg/dL, and elevated PSA at 35 ng/mL (Table 1). No prior PSA levels were available for comparison.

**Table 1:** Selected laboratory findings on presentation H: high (above reference range); WBC: white blood cells; BUN: blood urea nitrogen; PSA: prostate-specific antigen; HFP: high-power field

Laboratory Test	Parameters	Patient Values	Reference Range
Urinalysis	WBC	262 / HFP (H)	0-5 / HFP
Serum	WBC	13.5 x 10^9^/L (H)	4.5-11 x 10^9^/L
	BUN	28 mg/dL (H)	7-20 mg/dL
	Creatinine	1.9 mg/dL (H)	0.50-1.10 mg/dL
	PSA	35 ng/mL (H)	0-4 ng/mL

**Figure 1:**
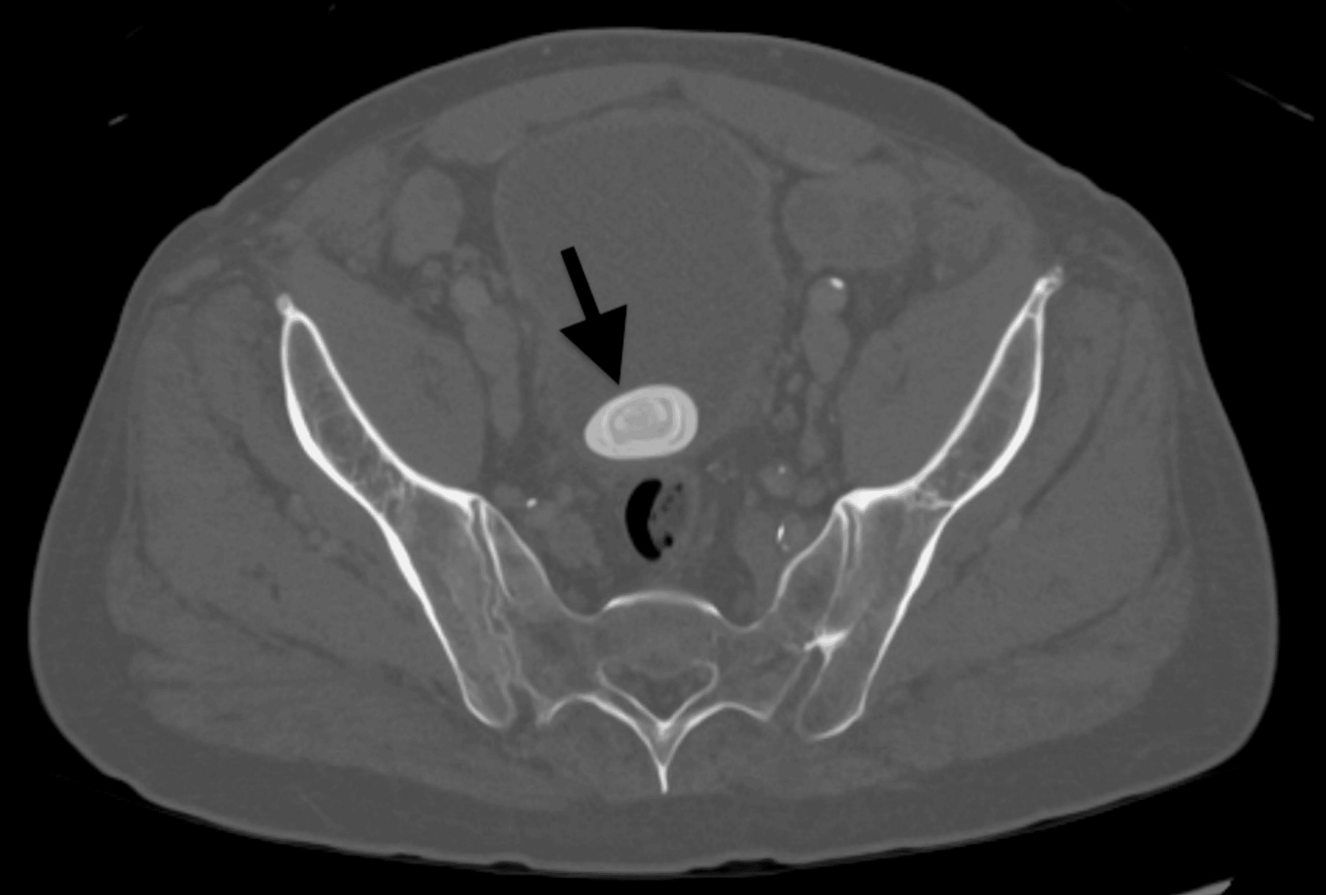
CT scan abdomen and pelvis with contrast (axial view) revealing a 3.5 cm bladder stone (arrow).

**Figure 2:**
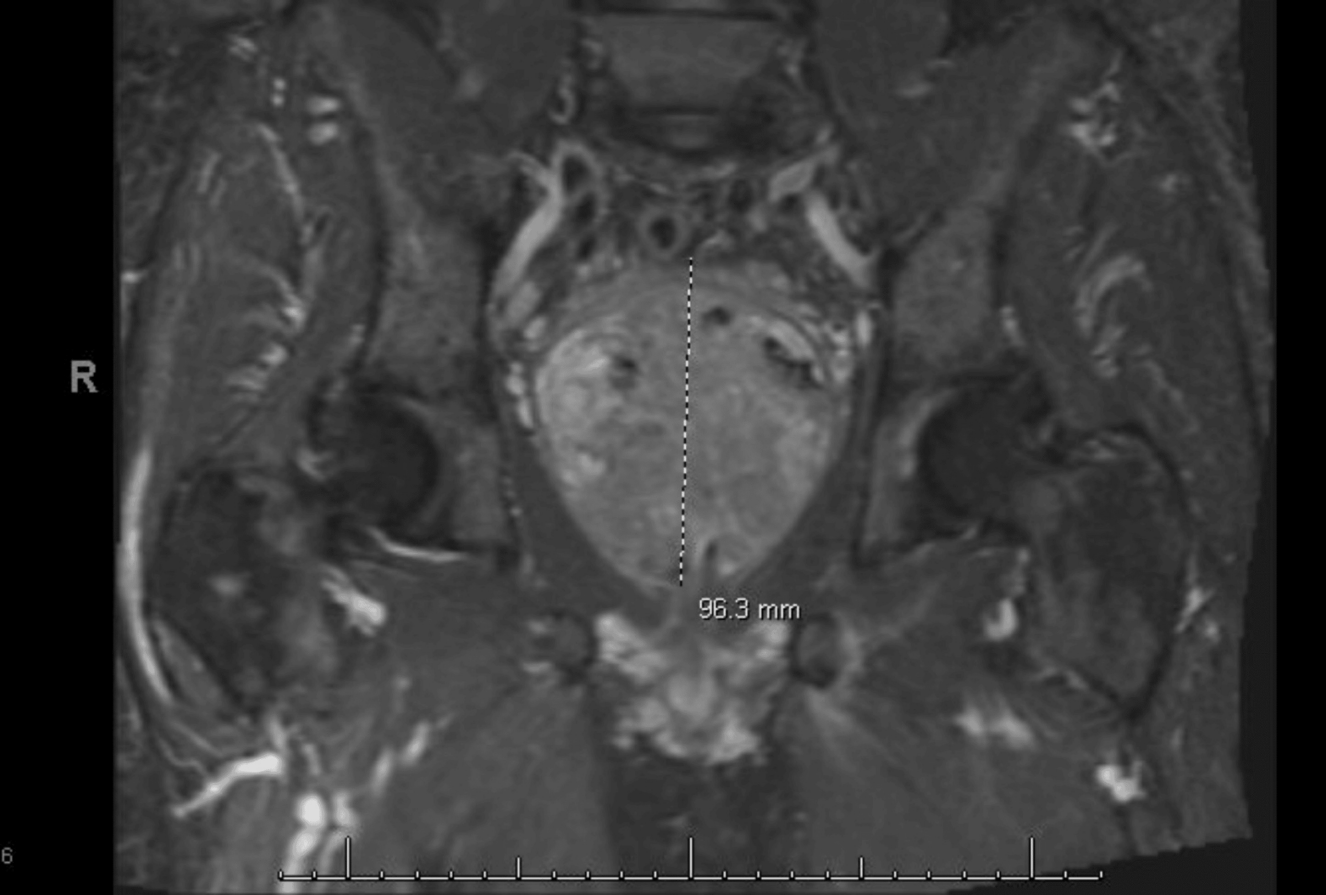
T2 STIR coronal MRI of the pelvis without contrast revealing prostatomegaly measuring 9.5 x 8.3 x 9.6 cm, with heterogeneous appearance. STIR: short tau inversion recovery

**Figure 3:**
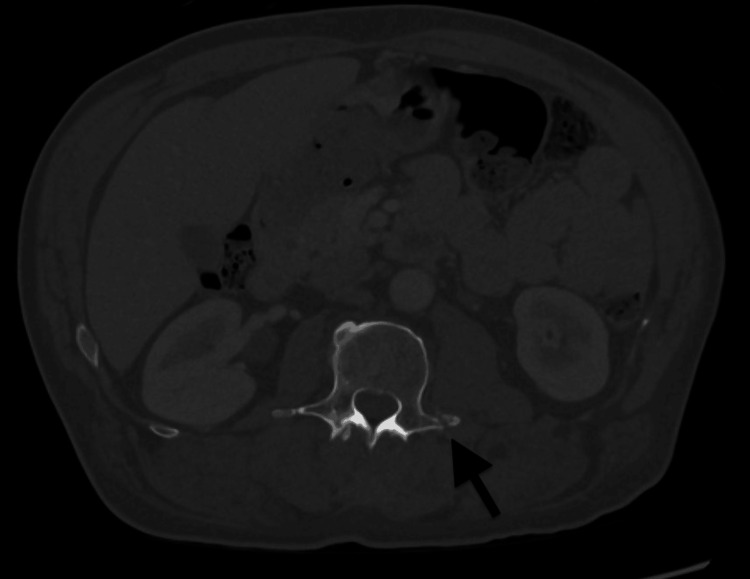
CT of the abdomen (axial view) revealing displaced non-acute appearing fractures of the left L2 and L3 transverse processes.

A computed tomography (CT) scan of his abdomen and pelvis was performed on the day of admission and revealed a 3.5 cm bladder stone and bladder wall thickening (Figure 1). The CT scan also revealed a 6.3 x 3.5 x 5.8 cm rim-enhancing fluid collection in the right inferior gluteal musculature. A short tau inversion recovery (STIR) T2 MRI was performed on the third day of admission to better identify fluid and edema and evaluate the gluteal collection. This sequence is preferred when highlighting fluid and inflammation. The MRI revealed significant prostate enlargement as an incidental finding, measuring 9-10 cm with extensive retroperitoneal and pelvic lymphadenopathy (Figure 2). The CT of the abdomen and pelvis also revealed fractures of the left L2 and L3 transverse processes with abnormal surrounding enhancement (Figure 3).

The patient improved with antibiotic treatment for his UTI as well as tamsulosin for his urinary tract obstruction. The gluteal collection was drained percutaneously via ultrasound-guided aspiration. Approximately 15 mL of dark red, blood-tinged fluid was aspirated, consistent with a hematoma. He was referred to urology for the bladder stone and prostatic enlargement with a plan for possible biopsy as an outpatient. He was also referred to an orthopedic spine team in the hospital, which suggested he remain in a thoracic lumbar sacral orthosis (TLSO) brace with no excessive bending, lifting, or twisting. After seven days in the hospital, he was discharged with resolution of the UTI and fecal incontinence. 

## Discussion

This case reveals an atypical presentation of a complicated UTI in an elderly male. The case was complicated by fecal incontinence, vertebral fractures, prior chronic catheterization, and a bladder stone. While these findings explained his risk factors for recurrent infection, concurrent prostatomegaly with an elevated PSA and lymphadenopathy raised suspicion for underlying malignancy. Severe infections may mimic the signs and symptoms of malignancy, creating significant diagnostic uncertainty and increasing the risk of anchoring bias early in the evaluation. Careful maintenance of a broad differential diagnosis is therefore essential to avoid premature diagnostic closure.

Catheter use is a common cause of UTIs, especially long-term catheter use. Despite advancements in prevention, catheter-associated UTIs remain a significant healthcare concern, and antibiotic resistance rates are alarmingly high. The abiotic surface of the catheter allows bacteria to be shielded from antibiotics, create resistance, and continue to colonize and multiply in the urinary tract. Because of the acceleration in antibiotic resistance, researchers are aiming to develop non-antibiotic strategies to prevent catheter-associated UTIs [5]. Until a more reliable prevention method can be established, chronic catheter use should include frequent catheter replacement to minimize bacterial growth [6]. Although the patient did not have a catheter in place at the time of evaluation, his history of chronic catheter use remains clinically significant, as prior long-term catheterization may predispose to persistent urinary tract vulnerability and increased risk of infection.

It has been suggested that fecal incontinence can often lead to UTIs, especially in elderly patients [7]. Determining the underlying cause of his fecal incontinence could lessen the risk of recurrent infections. One possible cause of fecal incontinence is excessive straining to urinate, which can result from bladder outlet obstruction caused by a bladder stone or an enlarged prostate. Bladder stones are hard masses made of minerals found in the urine. The most common causes for bladder stone formation are blockage of the urinary tract or nerve damage [8]. In some cases, small stones may pass on their own; however, in most cases, they require medical assistance for removal. Removal of the stone can involve a procedure that breaks the stone into smaller pieces for excretion, called cystolitholapaxy, which is generally a safe and effective outpatient procedure with little downtime [9].

The next step in addressing bladder outlet obstruction is investigating the enlarged prostate. The prostate measured 9.5 × 8.3 × 9.6 cm on imaging. Prostate volume was calculated using the ellipsoid formula, multiplying length, width, and height by 0.52, yielding approximately 395 mL in this case. This represents a significantly enlarged prostate compared with the normal adult range of 20-30 mL [10]. PSA levels and digital rectal examinations have been used as detection methods for prostate malignancy; however, they are not definitive. No prior PSA values were available, limiting the ability to determine whether the elevated PSA was chronic or acutely related to infection. Patients with suspected prostate cancer should undergo a standard transrectal ultrasound-guided biopsy for a definitive diagnosis [11]. Patients with localized prostate cancer may benefit from radical prostatectomy; however, perioperative morbidity and mortality are increased in elderly patients [12]. Patient selection according to age and current health status is important when deciding between surgery and a more conservative treatment plan. 

Another factor that may have caused his fecal incontinence and subsequent UTI was possible nerve damage from vertebral fractures. Fracture in the lumbar spine can affect both the lumbar and sacral plexus, resulting in impaired bladder and rectal control. As many as 77% of patients with a spinal cord injury experience fecal incontinence [13]. Any injury below T12 can increase a patient's risk for loss of sensation when the bowel is full. Due to the close proximity of the bladder and the bowel, functional interaction is very likely. Treatment should be tailored for symptom management, as the impact of bowel problems on a patient's quality of life can be devastating. Although the patient denied any recent falls, accidents, or trauma, his use of a walker with intermittent reliance on a wheelchair suggests an elevated fall risk. An unwitnessed or unreported fall prior to hospitalization remains a plausible explanation for the vertebral fractures and the gluteal hematoma.

The coexistence of bladder stone, UTI, and concerning prostate findings demonstrates the danger of diagnostic anchoring. Anchoring bias is defined as the focus on a single initial piece of evidence when making clinical decisions without taking later findings into account [14]. This case is notable because the coexistence of a bladder stone and infection provided a convincing explanation for his symptoms, yet additional findings of prostatomegaly, lymphadenopathy, vertebral fractures, and elevated PSA raised concern for possible malignancy. Anchoring bias is a well-documented contributor to diagnostic delay, which could prevent patients from receiving life-saving treatments [14]. In this patient, additional evaluation for possible prostate malignancy is warranted given his elevated PSA, enlarged prostate, and surrounding lymphadenopathy. This case demonstrates how coexisting pathology could be overlooked and can delay diagnosis and management. 

This patient’s condition appears to have progressed, at least in part, due to delayed medical attention or suboptimal prior management, allowing multiple pathologies to advance unchecked. His comorbidities likely exerted synergistic effects, with each contributing to the deterioration or exacerbation of others. Treating the homeless patient population provides challenges with follow-up and treatment adherence. A healthcare team with a community outreach focus, along with local and state agency involvement, is needed to ensure quality of care to help patients like this recover [15]. With assistance programs, individuals experiencing homelessness are better able to access medical treatments by receiving support with transportation, appointment scheduling, case management, and coverage for costs that would otherwise prevent access to care. 

## Conclusions

Clinicians must remain vigilant for dual pathology, as infection and obstruction may coexist with malignancy. Atypical features, such as fecal incontinence, spinal changes, or lymphadenopathy, should prompt reconsideration of the initial diagnosis to avoid anchoring bias. This case underscores how complex pathology can increase the risk of anchoring bias and highlights the importance of maintaining a broad differential in elderly men presenting with recurrent UTI.
